# Erratum to: The Bernese peri-acetabular osteotomy through a modified approach. A technical note

**DOI:** 10.1007/s11832-014-0582-3

**Published:** 2014-04-09

**Authors:** Paul Whittingham-Jones, Nirav K. Patel, Aresh Hashemi-Nejad

**Affiliations:** Royal National Orthopaedic Hospital (RNOH), Stanmore, Middlesex, HA7 4LP UK

## Erratum to: J Child Orthop (2013) 7:107–110 DOI: 10.1007/s11832-013-0485-8

In the original publication, the first and surname of the second author was wrongly published as “N. Kirit Patel” in affiliation. The correct name should read as “N. K. Patel”.

